# A nomogram based on clinicopathological and ultrasound characteristics to predict central neck lymph node metastases in papillary thyroid cancer

**DOI:** 10.3389/fendo.2023.1267494

**Published:** 2024-02-12

**Authors:** Fei Chen, Shuiping Jiang, Fan Yao, Yixi Huang, Jiaxi Cai, Jia Wei, Chengxu Li, Yanxuan Wu, Xiaolin Yi, Zhen Zhang

**Affiliations:** ^1^ General Surgery Center Department of Thyroid Surgery, Zhujiang Hospital, Southern Medical University, Guangzhou, Guangdong, China; ^2^ Endocrinology Department, Zhujiang Hospital, Southern Medical University, Guangzhou, Guangdong, China

**Keywords:** nomogram, ACR TI-RADS, model, lymph node metastasis, papillary thyroid carcinoma

## Abstract

**Purpose:**

Papillary thyroid cancer (PTC) has grown rapidly in prevalence over the past few decades, and central neck lymph node metastasis (CNLNM) is associated with poor prognoses. However, whether to carry out preventive central neck lymph node dissection (CNLND) is still controversial. We aimed to construct a prediction model of CNLNM to facilitate making clinical surgical regimens.

**Methods:**

A total of 691 patients with PTC between November 2018 and December 2021 were included in our study. Univariate and multivariate analyses were performed on basic information and clinicopathological characteristics, as well as ultrasound characteristics (American College of Radiology (ACR) scores). The prediction model was constructed and performed using a nomogram, and then discriminability, calibrations, and clinical applicability were evaluated.

**Results:**

Five variables, namely, male, age >55 years, clinical lymph node positivity, tumor size ≥1 cm, and ACR scores ≥6, were independent predictors of CNLNM in the multivariate analysis, which were eventually included to construct a nomogram model. The area under the curve (AUC) of the model was 0.717, demonstrating great discriminability. A calibration curve was developed to validate the calibration of the present model by bootstrap resampling, which indicated that the predicted and actual values were in good agreement and had no differentiation from the ideal model. The decision curve analysis (DCA) indicated that the prediction model has good clinical applicability.

**Conclusions:**

Our non-invasive prediction model combines ACR scores with clinicopathological features presented through nomogram and has shown good performance and application prospects for the prediction of CNLNM in PTCs.

## 1 Introduction

The prevalence of thyroid cancer has dramatically increased in the past few decades, among which papillary thyroid cancer (PTC) accounts for the largest proportion of approximately 80%–90% (1, 2). Although PTC has a great prognosis and a low risk, it has a high central neck lymph node metastasis (CNLNM) rate of approximately 20%–90%, which is closely related to the poor postoperative recurrence rate and survival outcome (3, 4). As we all know, central neck lymph node dissection (CNLND) facilitates reducing postoperative recurrence and avoiding a second surgery, but unnecessary prophylactic lymph node dissection may also lead to many other complications, such as permanent hypoparathyroidism and permanent laryngeal nerve damage (5). Accordingly, the prediction of CNLNM is critical for the accurate dissection of CNLNM, the formulation of a preoperative treatment plan, and postoperative personalized follow-up and management plan.

Ultrasound and computed tomography (CT) examination can predict CNLNM of thyroid cancer to a certain extent, but a recent meta-analysis indicated that the specificity and sensitivity were only 0.95 and 0.35, respectively, for ultrasound and 0.46 and 0.88, respectively, for CT in predicting CNLNM (6). Although fine-needle aspiration (FNA) cytology is the gold standard for the diagnosis of PTC and CNLNM (7), the lesion may not be obtained by puncture, and non-invasive examinations are more acceptable to patients. Accordingly, preoperative evaluation of CNLNM is still a crucial clinical challenge, and a new and high-efficiency prediction model is urgently needed to predict the risk of CNLNM.

Thyroid Imaging Reporting and Data System (TI-RADS), a hazard classification of thyroid nodules by scoring their characteristics, presented by the American College of Radiology (ACR) in 2017, provides greater accuracy in the diagnosis of PTC (8). Although the ACR score has been proposed to assess the malignancy of thyroid nodules, several researchers in recent years have suggested that the ACR score or ACR classification is an independent risk factor and can be used as a prediction model for CNLNM in PTC (9–13). For instance, Park et al. proposed that higher ACR TI-RADS scores were a predictor of CNLNM for small PTC (10–20 mm) (10). Nevertheless, all studies did not construct a nomogram when identifying risk factors for CNLNM by single- and multi-factor analyses.

According to our knowledge, this study is the first to construct a prediction model to draw a nomogram based on the ACR score for CNLNM. The purpose of this research is to develop a nomogram based on ACR score, cervical ultrasound features, and clinicopathological characteristics for the prediction of CNLNM in PTCs preoperatively and to guide clinical therapeutic regimen, surgical program, and postoperative follow-up plan.

## 2 Materials and methods

### 2.1 Patients and study design

This study is a retrospective research that was approved by the Clinical Research Center. All records between November 2018 and December 2021 were derived from Zhujiang Hospital, Southern Medical University, with permission of the research ethics committee and verbal consent of participants. A total of 691 patients enrolled in this research underwent thyroidectomy and CNLND and were diagnosed with PTC by pathology after surgery. Any participant who met the following criteria was excluded: 1) participants with a previous history of thyroid ablation or thyroid surgery; 2) participants with other malignant tumors, such as gastric cancer and medullary thyroid cancer; 3) participants with incomplete clinicopathological and ultrasound data; 4) patients with unclear ultrasound images and foci that cannot be diagnosed and evaluated.

### 2.2 Sociodemographic information

General information on the following was collected: age (<55 and ≥55 years), sex (female and male), and body mass index (BMI, <25 and ≥25 kg/m^2^).

### 2.3 Ultrasonography and ACR TI-RADS scoring procedure

A routine ultrasound examination was performed before surgery to measure the thyroid nodules and neck lymph nodes to determine the subsequent surgical plan. Ultrasonography for thyroid was performed using RESONA 70B (Mindray, Shenzhen, China) or GE Logiq 9, ARIETTA 850 (Hitachi, Tokyo, Japan) equipped with a linear array transducer in 5–20 MHz or 5–13 MHz. Ultrasound images were saved for subsequent data analysis. Two doctors who specialize in the endocrine system with at least 10 years of work experience retrospectively analyzed the images without knowledge of the basic and clinicopathological information, and disputes were resolved by consensus through discussion. The latest ultrasound images were included if the patient has several preoperative ultrasound images. The appearance of ≥2 suspected lesions in the thyroid was considered to be multifocal. The node with the highest ACR TI-RADS score was enrolled in this research in the case of multiple nodes. Clinically node-negative (cN0) PTC refers to the absence of all typical cervical ultrasound features, including microcalcifications, peripheral blood flow features, round lymph nodes, necrosis, unclear lymphatic portal structure, and focal hyperechoic. Diffuse lesions were confirmed by ultrasound reports, which may be chronic lymphocytic thyroiditis.

Ultrasound parameters of thyroid nodules included echo (very hypoechoic, 3 points; hypoechoic, 2 points; isoechoic or hyperechoic, 1 point; anechoic, 0 points), composition (solid or almost completely solid, 2 points; mixed solid and cystic, 1 point; spongiform, cystic, or almost completely cystic, 0 points), shape (taller-than-wide, 3 points; wider-than-tall, 0 points), echogenic foci (punctate echogenic foci, 3 points; peripheral calcifications, 2 points; macrocalcifications, 1 point; large comet-tail artifacts or none, 0 points), and margin (extra-thyroidal extension, 3 points; irregular or lobulated, 2 points; smooth, ill-defined, 0 points). All points were calculated using the 2017 ACR TI-RADS criteria (8).

### 2.4 Surgery program and pathological examination

All patients were subjected to total thyroidectomy or lobectomy combined with ipsilateral CNLND if the diagnosis of PTC was verified by preoperative FNA or intraoperative frozen pathology. Aside from this, bilateral central and lateral lymph node excision was carried out in cases of positive CNLNM in preoperative ultrasound, preoperative FNA, or intraoperative frozen pathology. FNA was not routinely performed before surgery. Postoperative thyroid and lymph node specimens were paraffin-embedded and used for H&E staining and immunohistochemical staining to clarify the pathological diagnosis. All postoperative specimens obtained were checked retrospectively by two licensed pathologists from Zhujiang Hospital, Southern Medical University. Pathological characteristics included tumor size, tumor pathological type, extrathyroidal extension (including invasion of capsular), multifocality, mutation status of BRAFV600E (mutant, wild type, and not performed), and CNLNM (areas and quantities). Diagnosis of Hashimoto’s thyroiditis was based on the pathology report. The bilateral tumor was described as the existence of cancer in the two thyroid lobes. The diameter of a node with the highest ACR score of multiple lesions was regarded as the maximum size.

### 2.5 Statistical analysis

Groups of CNLNM were divided according to the CNLNM status, and the basic and clinical characteristics were listed. Categorical variables are described as the frequency with percentage. Continuous variables are shown as the mean with standard deviation. Odds ratios and 95% confidence intervals (95% CIs) were initially used to describe the potential factors associated with CNLNM in the univariate analysis. Variables with an overall p < 0.05 in single-factor analysis were further enrolled in the multivariable analysis for independent predictor selection using the stepwise method. CNLNM was then predicted using a nomogram derived from the multivariate analysis. Then, this nomogram was evaluated using the receiver operating characteristic (ROC) curve, calibration curves, and decision curve analysis (DCA) developed from two internal cohorts (*n* = 691) produced by bootstrap resampling.

Statistical analyses were carried out using SAS 9.4 (SAS Institute Inc.) and R software (version 4.2.1, MathSoft Inc.). The p-value was based on two-sided tests. p < 0.05 was considered statistically significant.

## 3 Results

The study included 691 PTC patients, of which 377 (54.6%) had positive nodes by postoperative pathology. Further, 510 microcarcinomas were included, of which 218 (42.75%) developed CNLNM. In the CNLNM-positive group, 43 (11.4%) were older than 55 years, 117 (31.2%) were male, and 233 (62.3%) had a CN0. In the CNLNM group, 95 (25.2%) had a total ACR score greater than 10, and 266 (70.6%) had a score of 6 to 10, which was higher than that of the CNLNM-negative group (p < 0.001). The basic and clinicopathological features of the PTC are listed in **Table 1**.

**Table 1:** Clinicopathological and US characteristics of 691 patients with PTCs according to CNLNM.

Characteristics	CNLNM		p-Value
	Yes (*n* = 377)	No (*n* = 314)	
Sex			0.005
Female	117 (31.2)	68 (21.7)	
Male	258 (68.8)	246 (78.3)	
Age (years)			
≤55.0	334 (88.6)	255 (81.2)	0.006
>55.0	43 (11.4)	59 (18.8)	
BMI (kg/m^2^)			0.297
<25.0	254 (67.7)	220 (71.4)	
≥25.0	121 (32.3)	88 (28.6)	
CN0			<0.001
Yes	233 (62.3)	262 (84.0)	
No	141 (37.7)	50 (16.0)	
Hashimoto’s thyroiditis			1
Present	279 (74.6)	232 (74.60)	
Absent	95 (25.4)	79 (25.40)	
Tumor size (cm)			<0.001
<1	214 (56.8)	257 (82.4)	
≥1	163 (43.2)	55 (17.6)	
BRAFV600E mutation			0.043
Yes	300 (81.3)	270 (86.0)	
No	54 (14.6)	27 (8.6)	
NP	15 (4.1)	17 (5.4)	
Multifocality			0.005
Yes	123 (32.6)	72 (22.9)	
No	254 (67.4)	242 (77.1)	
Tumor position			0.412
Single lobe	179 (47.86)	140 (44.73)	
Bilateral lobe	195 (52.14)	173 (55.27)	
Bilateral tumor			0.017
Yes	93 (24.7)	54 (17.2)	
No	284 (75.3)	260 (82.8)	
Diffuse lesions			0.392
Yes	54 (14.4)	52 (16.8)	
No	321 (85.6)	258 (83.2)	
Extrathyroidal extension			0.001
Yes	137 (38.5)	83 (26.8)	
No	143 (61.5)	166 (73.2)	
ACR scores			<0.001
≤5	16 (4.2)	42 (13.4)	
6~10	266 (70.6)	210 (66.9)	
≥11	95 (25.2)	62 (19.7)	

US, ultrasound; CNLNM, central neck lymph node metastasis; BMI, body mass index; CN0, clinically node-negative; NP, not performed; ACR, American College of Radiology.

### 3.1 Univariable and multivariable analysis

Univariable and multivariable analyses were conducted to find out risk factors significantly associated with CNLNM (**Table 2**), including variables of sex, age, BMI, CN0, Hashimoto’s thyroiditis, tumor size, BRAFV600E mutation, multifocality, tumor position, bilateral tumor, diffuse disease, extrathyroidal extension, and ACR scores. The outcome of the univariable analysis suggested that age, sex, CN0, tumor size, BRAFV600E mutation, multifocality, bilateral tumor, extrathyroidal extension, and ACR scores were significantly correlated with CNLNM (all p < 0.05). A multivariable analysis was further performed, and it showed that ACR scores ≥6 (6 ≤ ACR scores ≤ 10: OR = 3.65, 95% CI: 1.80–7.39, p < 0.001; ACR scores ≥11: OR = 4.37, 95% CI: 2.04–9.36, p < 0.001), clinical lymph node positivity (OR = 2.74, 95% CI: 1.84–4.07, p < 0.001), tumor size ≥1 cm (OR = 3.23, 95% CI: 2.20–4.74, p < 0.001), age > 55 years (OR = 0.52, 95% CI: 0.32–0.84, p = 0.008), and male (OR = 1.64, 95% CI: 1.12–2.41, p = 0.012) were independent risk factors of CNLNM (**Table 2**).

**Table 2:** Univariate analysis and multivariate analysis of factors associated with CNLNM in patients with PTC.

Characteristics	Univariable		Multivariable	
	OR (95% CI)	p-Value	OR (95% CI)	p-Value
Sex				
Female	1		1	
Male	1.64 (1.16–2.32)	0.005	1.64 (1.12–2.41)	0.012
Age (years)				
≤55.0	1		1	
>55.0	0.56 (0.36–0.85)	0.007	0.52 (0.32–0.84)	0.008
BMI (kg/m^2^)				
<25.0	1			
≥25.0	1.19 (0.86–1.65)	0.297	–	
CN0				
Yes	1		1	
No	3.17 (2.20–4.58)	<0.001	2.74 (1.84–4.07)	<0.001
Hashimoto’s thyroiditis				
Present	1			
Absent	1 (0.71–1.41)	1	–	
Tumor size (cm)				
<1	1		1	
≥1	3.56 (2.50–5.08)	<0.001	3.23 (2.20–4.74)	<0.001
BRAFV600E mutation				
Yes	0.56 (0.34–0.91)	0.019		
No	1		ns	
NP	0.44 (0.19–1.02)	0.055		
Multifocality				
Yes	1.63 (1.16–2.29)	0.005		
No	1		ns	
Tumor position				
Single lobe	1		–	
Bilateral lobe	0.88 (0.65–2.29)	0.412		
Bilateral tumor				
Yes	1.58 (1.08–2.29)	0.017	ns	
No	1			
Diffuse lesions				
Yes	0.84 (0.55–1.26)	0.393	–	
No	1			
Extrathyroidal extension				
Yes	1.71 (1.23–2.38)	0.001	ns	
No	1			
ACR scores				
≤5	1		1	
6~10	3.33 (1.82–6.08)	<0.001	3.65 (1.80–7.39)	<0.001
≥11	4.02 (2.08–7.77)	<0.001	4.37 (2.04–9.36)	<0.001

–, not involved in the multivariable analysis; ns, not significant; CNLNM, central neck lymph node metastasis; PTC, papillary thyroid carcinoma; BMI, body mass index; CN0, clinically node-negative; NP, not performed; ACR, American College of Radiology.

### 3.2 Establishment and validation of the nomogram

**Figure 1:**
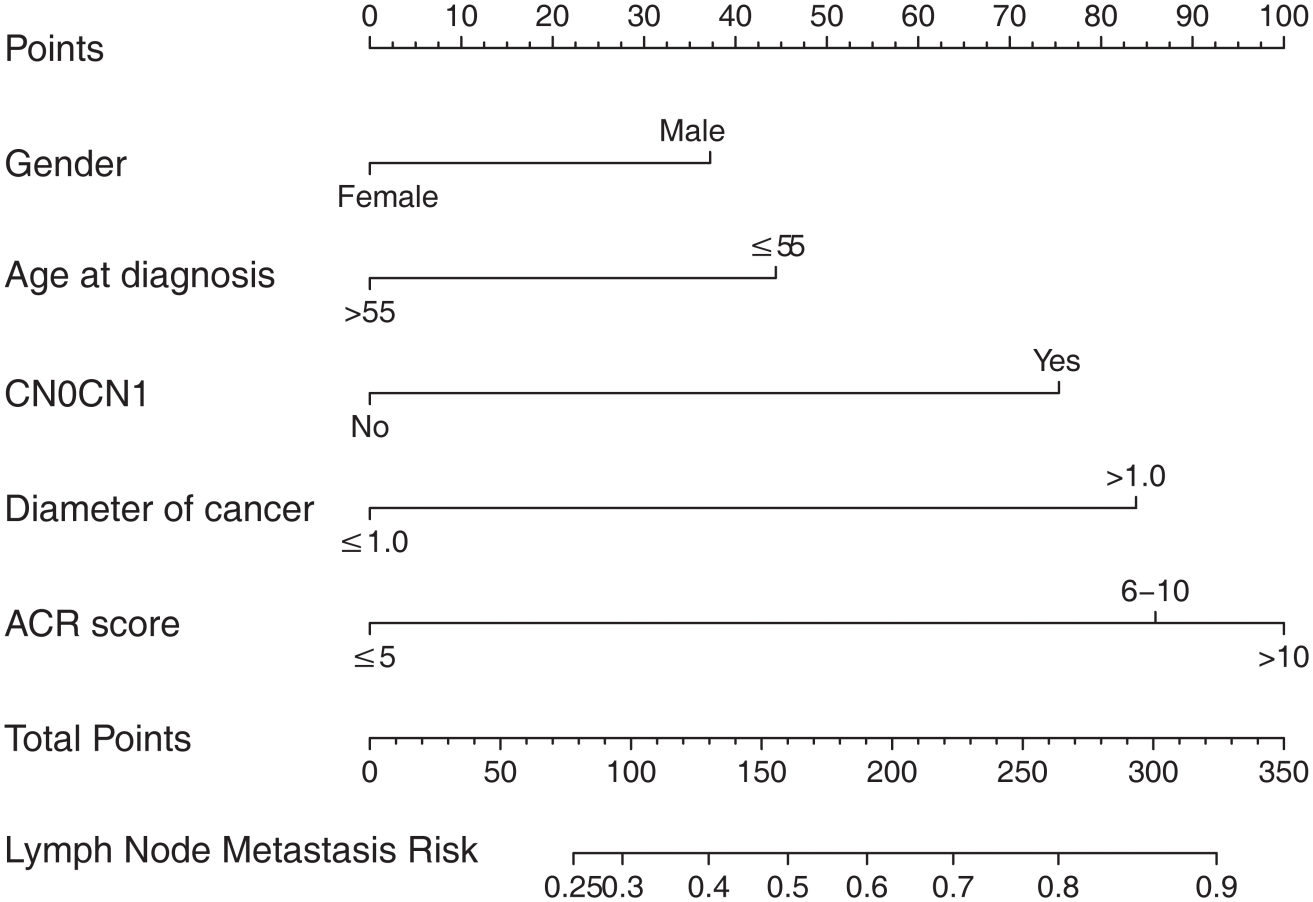
The nomogram for predicting CNLNM in patients with PTC. CNLNM, central neck lymph node metastasis; PTC, papillary thyroid carcinoma; CN0, Clinically node-negative.

**Figure 2:**
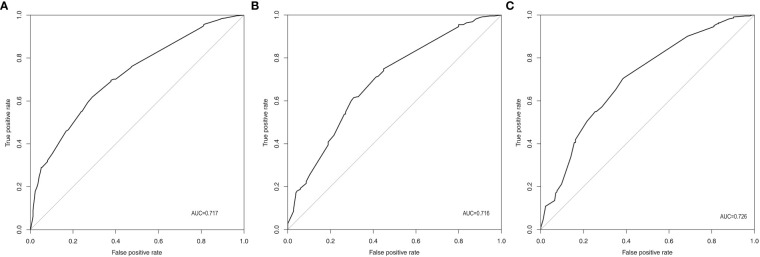
ROC of the prediction model for CNLNM. **(A)** Original training cohort (n=691), AUC=0.717. **(B)** First validating cohort from resampling bootstrap analysis (n=691), AUC=0.716. **(C)** Second validating cohort resampling bootstrap analysis (n=691), AUC=0.726. ROC, receiver-operating characteristic; CNLNM, central neck lymph node metastasis; AUC, area under the ROC curve.

**Figure 3:**
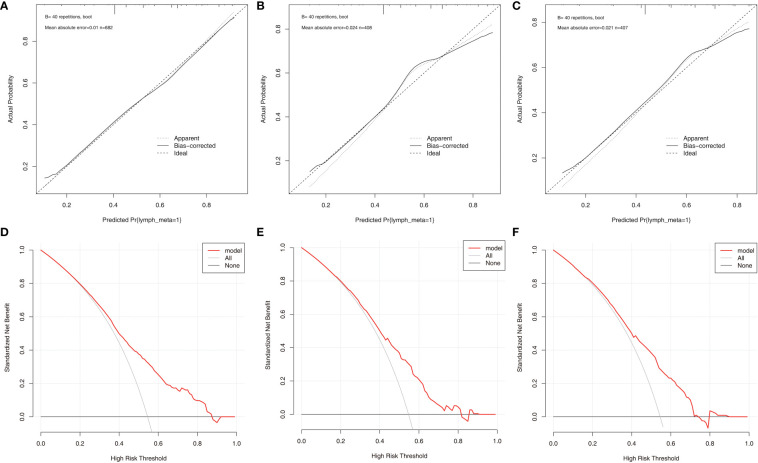
The calibration curves and DCA curves for the nomogram. **(A)** calibration curves in the training cohort. **(B)** calibration curves in the first validation cohort. **(C)** calibration curves in the second validation cohort. **(D)** The DCA in the training cohort. **(E)** The DCA in the first validation cohort. **(F)** The DCA in the second validation cohort. DCA, Decision curve analysis.

According to the final results of multivariable analysis, variables of age, sex, tumor size, CN0, and ACR score were eventually included to develop an easy-to-understand nomogram model (**Figure 1**), which could facilitate the prediction of CNLNM in PTCs. The area under the curve (AUC) of the ROC was 0.717 (**Figure 2A**). The established nomogram model was further evaluated using two independent cohorts produced by resampling bootstrap analysis, and the AUC values were 0.716 and 0.726 (**Figures 2B, C**). Three calibration curves were also developed to validate the calibration of the present model by bootstrap resampling (**Figure 3**). Great concordance was shown in the curves (ideal, apparent, and bias-corrected lines) derived from the training model (mean absolute error (MAE) = 0.010, **Figure 3A**) and two internal validation cohorts (MAE = 0.018 in **Figure 3B**; MAE = 0.019 in **Figure 3C**). The DCA was further conducted to evaluate the clinical practicability of the model in predicting CNLNM. The DCA curves showed that when the threshold probability of three cohorts was between 0.2 and 0.9, our prediction model was better than a no-treat or all-treat regimen.

## 4 Discussion

The increasing incidence of thyroid cancer, as we all know, is partly due to the advances in technology that allow for more accurate screening techniques now (14). However, along with the increased detection rates that now exist, some unnecessary surgeries have increased, and the resulting postoperative complications, such as hypothyroidism, hypoparathyroidism, and damage to the cervical laryngeal nerve, have been shown to cause lifelong inconvenience to patients (15–19). Hence, a judgment of lymph node status is essential for the decision of CNLND.

After reviewing previous studies that assessed risk factors for predicting CNLNM in PTCs, it was found that only a small number of studies have used total ACR scores. Different from previous guidelines, the 2017 ACR TI-RADS guidelines provide detailed scoring for each node in addition to classifying the nodes (TR1-TR5) only. A study suggested that an increased ACR TI-RADS score was related to CNLNM in both TR5 (diameter of tumor ≥10 mm) PTCs and TR4 PTCs13. Several other articles also believe that the ACR score is an independent risk factor of CNLNM10–12. However, none of them developed an easy-to-understand nomogram for the prediction model. From this, a nomogram based on ACR score, cervical ultrasound characteristics, and clinicopathological characteristics was established in the present study.

Risk factors of CNLNM were evaluated by single-factor and multivariate analyses in PTCs in our research. Sex, age, CN0, tumor size, BRAFV600E mutation, multifocality, bilateral tumor, extrathyroidal extension, and ACR scores were correlated with CNLNM in the univariate analysis. ACR scores ≥6 (6 ≤ ACR scores ≤ 10, p < 0.001; ACR scores ≥11, p < 0.001), clinical lymph node positivity (p < 0.001), age > 55 years (p = 0.008), female (p = 0.012), and tumor size ≥1 cm (p < 0.001) were independent risk factors of CNLNM.

In our study, ACR scores were divided into three groups (ACR scores ≤ 5, 6 ≤ ACR scores ≤ 10, and ACR scores ≥ 11), and in the multifactorial regression analysis, we found that ACR scores of 6–10 and ACR scores ≥11 were independent predictors for CNLNM for PTCs. We can also consider an ACR of 6–10 as 3.65 times and an ACR ≥ 11 as 4.37 times the risk of CNLNM in the group (ACR ≤ 5). Therefore, for PTC patients with an ACR score of ≥6, we should increase the emphasis on preoperative screening of cervical lymph nodes and encourage more preoperative examinations to help surgeons more accurately identify metastatic lymph nodes preoperatively for a more scientific surgical protocol, and more active follow-up is also needed.

Some studies concluded that tumor size ≥1 cm is more likely to develop CNLNM than tumor size <1 cm (20–22), although several studies have not segmented tumors by 1 cm (23–29), and some studies concluded that larger tumor volume is more likely to develop CNLNM (30, 31). In sum, CNLNM is generally more likely to occur with larger tumors. In our research, we discovered that tumor size ≥1 cm was an independent risk factor of CNLNM, which is consistent with previous findings (20–22). It is possible that the larger the tumor diameter, the more advanced the tumor growth, so CNLNM is more likely to occur. However, even if the tumor size is small, CNLNM may occur due to its strong invasiveness (25). We can only confirm the aggressiveness of the tumor by postoperative pathology at present, so we should put importance on the preoperative assessment of thyroid nodules and CNLNM regardless of the size of the tumor.

Many studies suggested that younger-age patients are more likely to develop CNLNM (20–22), and in line with these previous studies, we also found that patients aged <55 years are more likely to develop CNLNM, which may be due to the rapid rate of cell growth and renewal in younger people. However, a study concluded that age < 55 years is not a risk factor for central lymph node metastasis in PTC (32). Therefore, the relationship between CNLNM and age still needs more verification by more studies.

Although previous studies have suggested that women are more likely to develop thyroid cancer than men, some experts believe that this is due to female estrogen (33). Nevertheless, men are more likely to develop CNLNM once their immune defenses have been breached. The male sex was believed to be an independent risk factor in the previous research (20–23). However, the differences in the extent of thyroid cancer in men versus women may not only be related to biological factors. Several studies pointed out that a higher rate of CNLNM in men can also be attributed to delayed presentation and delayed surgery in men, as body habitus and neck size in men always lead to less palpable lymph nodes than in women (34–39). In our research, we also found that men were more likely to develop CNLNM.

For easier application and visualization of the results of predicting CNLNM risk, an easy-to-use nomogram based on ACR scores and clinicopathological characteristics that we screened out was constructed, which allowed us to quantify the impact of multiple independent variables on the prediction model. Interestingly, the ACR score in our predictive nomogram was the largest contributor to the total score. The nomogram is used as follows: first, draw a line through the values of the variables perpendicular to the axis of points and find the spot of each variable on it. Second, sum the points of all the variables to obtain the total points. Finally, find the spot of the total point axis and draw a line perpendicular to the risk axis, which is the risk of the CNLNM. Based on the nomogram, the AUC and C-index were 71.7% and 0.717, respectively, and the AUC values of two independent cohorts produced by resampling bootstrap analysis were 0.716 and 0.726, which demonstrated the great discriminability of this present prediction model. Calibration curves developed from the training model and two internal validation cohorts showed a good concordance between the predicted value and the actual value and no differentiation from the ideal model. The DCA curves showed great clinical practicability of the model in predicting CNLNM.

Although we obtained a prediction model with good performance, there are still several limitations. First, the present study was single-center retrospective research, and inevitably, confounding factors were present. More multicenter prospective studies are therefore required in the future. Second, since ultrasound features rely heavily on the experience of the doctor, while our ultrasound images are assessed by an experienced thyroid surgeon, subjective bias was inclined to occur, and thus, the objectivity of the findings was easily compromised. Additionally, it is well known that CNLNM is more prone to metastasis and not easily detected by ultrasound due to the interference of the thyroid gland and tracheal cartilage, so the present study only analyzed CNLNM due to the limited number of cases with lateral neck lymph node metastasis. Thus, neck lymph node metastasis in the lateral region demands more analysis urgently in the future. Finally, although our model has demonstrated good diagnostic performance through internal validation by bootstrapping analysis, overfitting of the model may also have occurred, and further external validation is still required.

## 5 Conclusions

Our model is a non-invasive prediction tool that combines ACR scores with clinicopathological features presented through nomogram, which can predict CNLNM in PTCs and has shown good performance and prospects for application.

## Data availability statement

The raw data supporting the conclusions of this article will be made available by the authors, without undue reservation.

## Ethics statement

The studies involving humans were approved by Research Ethics Committee of Zhujiang Hospital, Southern Medical University. The studies were conducted in accordance with the local legislation and institutional requirements. Written informed consent for participation was not required from the participants or the participants’ legal guardians/next of kin in accordance with the national legislation and institutional requirements.

## Author contributions

FC: Conceptualization, Data curation, Formal analysis, Funding acquisition, Writing – review & editing. SJ: Conceptualization, Methodology, Writing – original draft, Writing – review & editing. FY: Data curation, Writing – review & editing. YH: Data curation, Writing – review & editing. JC: Data curation, Writing – review & editing. JW: Data curation, Writing – review & editing. CL: Data curation, Writing – review & editing. YW: Data curation, Writing – review & editing. XY: Conceptualization, Writing – review & editing. ZZ: Conceptualization, Writing – review & editing, Supervision.
